# Supplementary material for: Delivering Positive Newborn Screening Results: Cost Analysis of Existing Practice versus Innovative, Co-Designed Strategies from the ReSPoND Study
Source: Int J Neonatal Screen. 2022 Mar 14;8(1):19. doi: 10.3390/ijns8010019 (PMC8951105; doi:10.3390/ijns8010019)
Supplement: Supplementary file 1 [file IJNS-08-00019-s001.zip › Figure S1 Existing pathways and implementation of co-designed interventions for models of care based on home-visits.pdf]

Figure S1. Existing pathways and implementation of co-designed interventions for models of care based on home-visits.

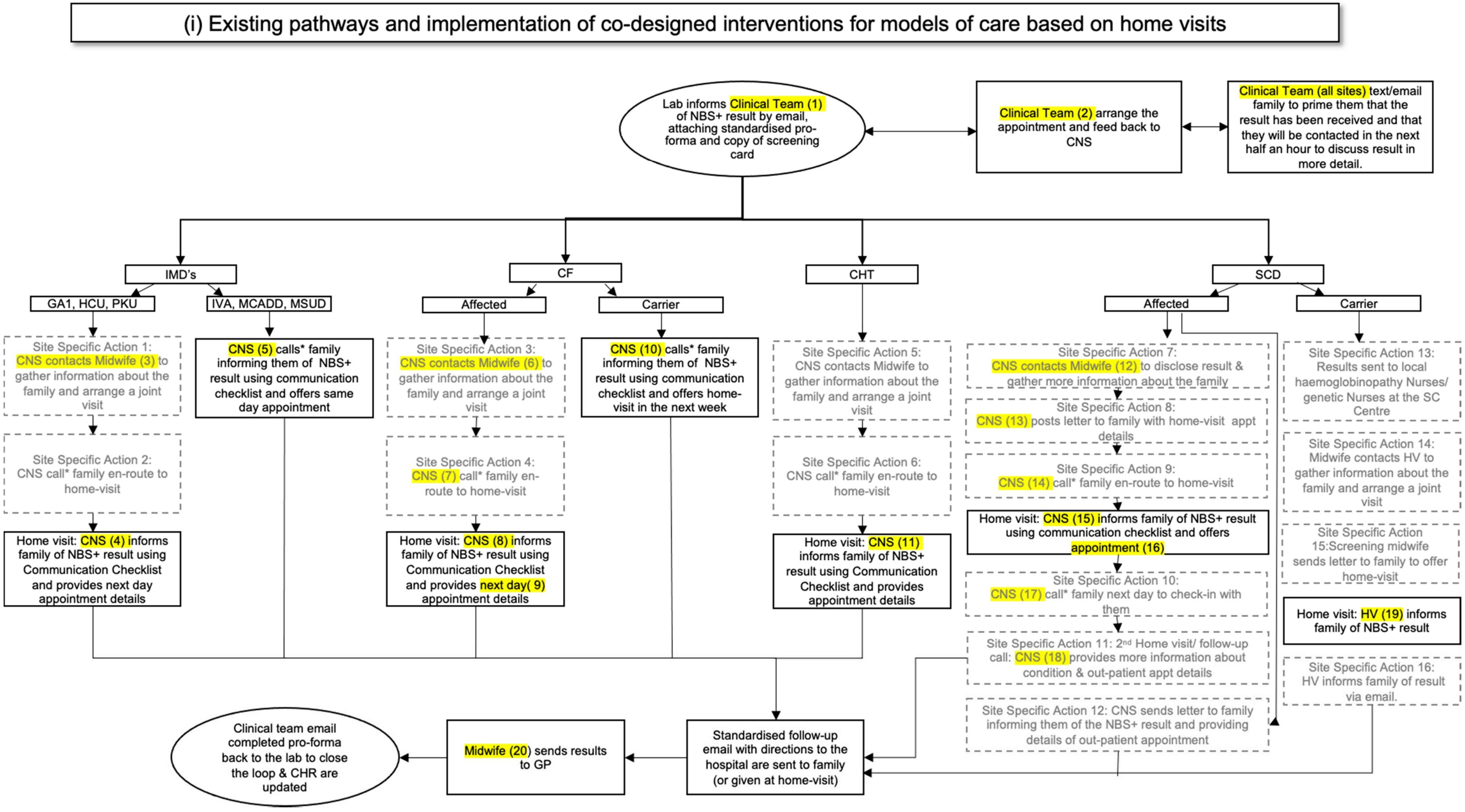

Figure S1 legend:
*Call refers to telephone call or video conference call where possible.

**Boxes with a grey dashed outline represent site specific actions, outlined below:**
Action 1: Specific to Site 1 & Site 9.<br>Action 2: Specific to Site 1 only.<br>Action 3: Specific to Sites 1, 2, 3, 7, 9, 10, 11, 13<br>Action 4: Specific to Sites 1, 3, 7, 11, 13<br>Action 5: Specific to Site 1 only.<br>Action 6: Specific to Site 1 only.<br>Action 7: Specific to Sites 1, 7, 9, 10.<br>Action 8: Specific to Site 6a and Site 9.<br>Action 9: Specific to Sites 1, 10, 11, 13.<br>Action 10: Specific to Sites 6a, 9, 10, 13.<br>Action 11: Specific to Site 6a 7, 10.<br>Action 12: Specific to Site 2 only.<br>Action 13: Specific to Site 1 only.<br>Action 14: Specific to Site 10 only.<br>Action 15: Specific to Site 12 only.<br>Action 16: Specific to Site 3 only.

Terms highlighted in yellow indicate that the designated clinical team member/timing varies depending on site. These are specified for each site below:
(1) CF Team (Site 2 CF only, Site 10 CF only, Site 13 CF only, Site 9 CF Affected only); CHD (Site 7 CF Carrier only); CNS (Sites 1, 7, 9, 13 SCD only); CNS & CP (Sites 3 and 13 IMDs only); CNS & HV (Site 8); CNS & HV Carrier Link (Site 7); CP (Site 11 MUD/IVA/GA1/HCU only, Site 10 CHT only, Site 8 CHT only, Site 1 CHT only, Site 10 CHT only); Endocrine Team (Site 7 CHT only); Haemoglobinopathy Counsellor & CP (Site 8 SCD only); Metabolic Nurse (Site 9 IMDs only); Midwife (Site 10 SCD only); Midwife & Endocrine Registrar (Site 9 CHT only); Midwife & IMD Team (Site 7 IMDs only); Screening Link HV (Sites 7 and 9 SCD & CF Carrier only); Screening Nurse (Site 11); Sickle Cell Centre (Site 9 SCD Affected only).<br>(2) Lab & Pathway Coordinator (Sites 7 and 9 CHT only); Clinical Team (all other sites).<br>(3) CNS contacts Community Midwife (Site 9); CNS contacts HV/Midwife (Site 1).<br>(4) CNS/Community Midwife (Site 9); CNS/HV/Midwife (Site 1).<br>(5) CNS (Site 1); CP (Site 9).<br>(6) CNS contacts CP (Sit 13); CNS contacts Midwife/HV (Site 1); CNS contacts HV (Sites 2,3,10); CNS contacts Screening Link HV (Sites 7 and 9); Screening Nurse contacts CNS (Site 11).<br>(7) CNS (Sites 3 and 13); HV Carrier Link (Site 7); Screening Nurse (Site 11).<br>(8) CF HV (Site 10); CNS (Site 3); CNS & HV (Sites 1 and 8); HV Screening Link (Site 9).<br>(9) Same day (Site 2); Next day (all other sites).<br>(10) CNS (Site 1); HV (Site 13); HV & CF Nurse (Site 2); HV Link & Family HV (Site 7).<br>(11) CNS & Midwife/HV (Site 1); Midwife (Site 8); Midwife/HV (Sites 7 and 9); GP Member (Sites 10).<br>(12) CNS contacts Midwife/HV (Site 1); CNS contacts CP (Site 11); CNS contacts Screening Midwife (Site 7); Homecare Practitioner contacts HV (Site 9); Midwife contacts HV (Site 10).<br>(13) CNS (KCH); Homecare Practitioner (Site 9).

(14) CNS (Sites 1 and 13 & Sheffield); HV/Midwife (Site 10); Screening Nurse (Site 11).

(15) CNS (Sites 1, 6a, 7, 11, 13); Homecare Practitioner (Site 9); HV/Midwife (Site 10).

(16) Hospital Appointment (Sites 1 and 10); Next day follow-up home-visit (Site 7); 2-week follow-up home-visit (Site 6a).

(17) CNS (Sites 6a and 13); Homecare Practitioner (Site 9); Midwife (Site 10).

(18) CNS (Sites 6a and 7); Midwife (Site 10).

(19) HV (Site 2); Midwife/HV (Sites 8 and 10); Screening Link HV (Site 9); Screening Link HV & Family HV (Site 7); Screening Midwife (Site 12);

(20) Biochemist (Site 3); CP/CNS (Sites 1, 5, 7, 8, 10 CF only, Sites 12 and 13); Consultant (Site 10 IMDs only); Homecare Practitioner (Site 9 SCD only): Lab (Sites 2, 4, 7, 10 CHT only); Midwife (Site 10 SCD only); Screening Nurse (Site 11).

Abbreviations:

Clinical Nurse (CNS)

Clinical Psychologist (CP)

General Practitioner (GP)

Health Visitor (HV)
