# Supplementary material for: Delivering Positive Newborn Screening Results: Cost Analysis of Existing Practice versus Innovative, Co-Designed Strategies from the ReSPoND Study
Source: Int J Neonatal Screen. 2022 Mar 14;8(1):19. doi: 10.3390/ijns8010019 (PMC8951105; doi:10.3390/ijns8010019)
Supplement: Supplementary file 1 [file IJNS-08-00019-s001.zip › Figure S2 Existing pathways and implementation of co-designed interventions for models of care based on telecommunications.pdf]

Figure S2. Existing pathways and implementation of co-designed interventions for models of care based on telecommunications.

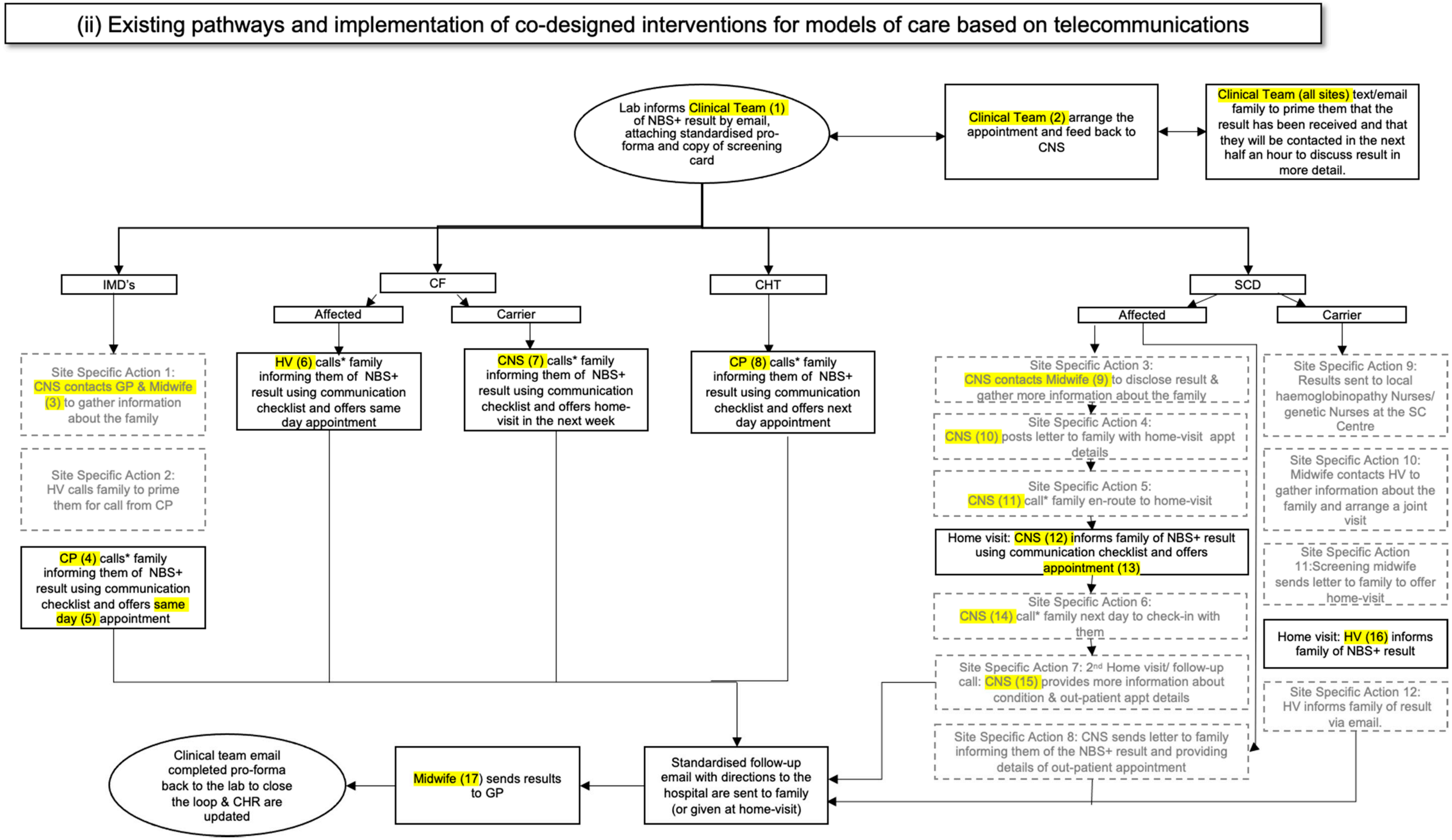

Figure S2 legend:

***Call refers to telephone call or video conference call where possible.**

**Boxes with a grey dashed outline represent site specific actions, outlined below:**
Action 1: Specific to Sites 2, 10, 11, 13.<br>Action 2: Specific to Site 2 only.<br>Action 3: Specific to Sites 1, 9, 10, 11.<br>Action 4: Specific to Sites 6a and 9.<br>Action 5: Specific to Sites 1, 10, 11, 13.<br>Action 6: Specific to Sites 6a, 9, 10, 13.<br>Action 7: Specific to Sites 6a, 7, 10.<br>Action 8: Specific to Site 2 only.<br>Action 9: Specific to Site 1 only.<br>Action 10: Specific to Site 10 only.<br>Action 11: Specific to Site 12 only.<br>Action 12: Specific to Site 3 only.

Terms highlighted in yellow indicate that the designated clinical team member/timing varies depending on site. These are specified for each site below:
(1) CF Team (Site 12 CF Affected only); CNS & CP (Sites 6a, 5, 6, 13); CP (Sites 11, 12, 13 CHT only, Site 4 CHT only, Site 2, 3, 10 IMDs only); Midwife & IMD Team (Site 7 IMDs only); Nurse Practitioner (Site 5 IMDs only); Screening Coordinator (Site 11 CHT only); Screening Midwife (Site 12 SCD & CF Carrier only).<br>(2) Screening Coordinator (Site 11 CHT only); Clinical Team (all other sites).<br>(3) CNS contacts GP & Midwife (Site 13); CP contacts GP (Site 10); CP contacts Midwife and HV (Site 2).<br>(4) CP/CNS depending on complexity (Site 6); CNS (Sites 5 and 13); CP (Sites 2 and 10); Midwife (Site 7); Screening Nurse (Site 11).<br>(5) Same day appointment (IVA/MCADD/MSUD); Next day appointment (PKU/GA1/HCU).<br>(6) CNS (Site 5, Portsmouth); CP (Site 6a local babies); HV (Site 6a remote babies).<br>(7) CNS (Site 5); CP/HV (KCH); Screening Midwife (Site 12).<br>(8) CP or Endocrine Nurse Specialist (Site 2); CP (Sites 3 and 13); CP or Screening Midwife (Site 12); GP or CNS (Site 5); Screening Coordinator/Midwife (Site 11)<br>(9) CNS contacts Midwife/HV (Site 1); CNS contacts CP (Site 11); CNS contacts Screening Midwife (Site 7); Homecare Practitioner contacts HV (Site 9); Midwife contacts HV (Site 10).<br>(10) CNS (Site 6a); Homecare Practitioner (Site 9).<br>(11) CP or Endocrine Nurse Specialist (Site 2); CP (Sites 3 and 13); CP or Screening Midwife (Site 12); GP or CNS (Site 5); Screening Coordinator/Midwife (Site 11)<br>(12) CNS (Sites 1, 6a, 7, 11, 13); Homecare Practitioner (Site 9); HV/Midwife (Site 10).<br>(13) Hospital Appointment (Sites 1 and 10); Next day follow-up home-visit (Site 7); 2-week follow-up home-visit (Site 6a).<br>(14) CNS (Sites 6a and 13); Homecare Practitioner (Site 9); Midwife (Site 10).<br>(15) CNS (Site 6a and 7); Midwife (Site 10).<br>(16) HV (Site 2); Midwife/HV (Sites 8 and 10); Screening Link HV (Site 9); Screening Link HV & Family HV (Site 7); Screening Midwife (Site 12);

(17) Biochemist (Site 3); CP/CNS (Sites 1, 5, 7, 8, 10 CF only, Sites 12 and 13) Consultant (Site 10 IMDs only); Homecare Practitioner (Site 9 SCD only): Lab (Sites 2, 4, 7, 10 CHT only); Midwife (Site 10 SCD only); Screening Nurse (Site 11).

Abbreviations:

Clinical Nurse (CNS)

Clinical Psychologist (CP)

General Practitioner (GP)

Health Visitor (HV)
