# Supplementary material for: Delivering Positive Newborn Screening Results: Cost Analysis of Existing Practice versus Innovative, Co-Designed Strategies from the ReSPoND Study
Source: Int J Neonatal Screen. 2022 Mar 14;8(1):19. doi: 10.3390/ijns8010019 (PMC8951105; doi:10.3390/ijns8010019)
Supplement: Supplementary file 1 [file IJNS-08-00019-s001.zip › Table S2 IP home-visit- RESPOND (blinded).pdf]

Table S2. Number of contacts and relative cost per each centre – home-visit pathway.

Centre 1

Number of contacts

	Inherited metabolic disorders (IMD)										
	**medium chain acyl CoA dehydrogenase deficiency (MCADD)**	**maple syrup urine disease (MSUD)**	**isovaleric aciduria (IVA)**	**glutaric aciduria type 1 (GA1)**	**phenylketonuria (PKU)**	**homocystinuria (HCU)**	**Hypothyroidism (CHT)**	**Sickle Cell Disorder (SCD)**		**Cystic Fibrosis (CF)**	
**Personnel - Clinical path items**								NBS+: Affected	NBS+: Carrier	NBS+: Affected	NBS+: Carrier
CNS - Clinical nurse specialist											
home visit	1	1	1	1	1	1	1	1	1	1	2
surgery/hospital visit	1	1	1	1	1	1	1	1		1	
not face-to-face external interaction	1	1	1	1	1	1	2	2	2	2	1
not face-to-face internal interaction	3	3	3	3	3	3	4	4	2	4	2
Health visitor/midwife											
home visit				0	0	0	0	0		0	
surgery/hospital visit											
not face-to-face external interaction											
not face-to-face internal interaction				0	0	0	0	0		0	
Consumables											
Leaflet											
GP											
home visit											
surgery/hospital visit											
not face-to-face external interaction											
not face-to-face internal interaction	1	1	1	1	1	1	1	1	1	1	1
Consultant (hospital based)											
home visit											
surgery/hospital visit	1	1	1	1	1	1	1	1		1	
not face-to-face external interaction											
not face-to-face internal interaction											

Cost

	Inherited metabolic disorders (IMD)						Hypothyroidism (CHT)	Sickle Cell Disorder (SCD)		Cystic Fibrosis (CF)	
	medium chain acyl CoA dehydrogenase deficiency (MCADD)	maple syrup urine disease (MSUD)	isovaleric aciduria (IVA)	glutaric aciduria type 1 (GA1)	phenylketonuria (PKU)	homocystinuria (HCU)					
**Personnel - Clinical path items**								NBS+: Affected	NBS+: Carrier	NBS+: Affected	NBS+: Carrier
CNS - Clinical nurse specialist											
home visit	£104	£104	£104	£104	£104	£104	£104	£104	£104	£104	£208
surgery/hospital visit	£65	£65	£65	£65	£65	£65	£65	£65	£0	£65	£0
not face-to-face external interaction	£27	£27	£27	£27	£27	£27	£54	£54	£54	£54	£27
not face-to-face internal interaction	£80	£80	£80	£80	£80	£80	£107	£107	£54	£107	£54
Health visitor/midwife											
home visit	£0	£0	£0	£0	£0	£0	£0	£0	£0	£0	£0
surgery/hospital visit	£0	£0	£0	£0	£0	£0	£0	£0	£0	£0	£0
not face-to-face external interaction	£0	£0	£0	£0	£0	£0	£0	£0	£0	£0	£0
not face-to-face internal interaction	£0	£0	£0	£0	£0	£0	£0	£0	£0	£0	£0
Consumables											
Leaflet	£0	£0	£0	£0	£0	£0	£0	£0	£0	£0	£0
GP											
home visit	£0	£0	£0	£0	£0	£0	£0	£0	£0	£0	£0
surgery/hospital visit	£0	£0	£0	£0	£0	£0	£0	£0	£0	£0	£0
not face-to-face external interaction	£0	£0	£0	£0	£0	£0	£0	£0	£0	£0	£0
not face-to-face internal interaction	£87	£87	£87	£87	£87	£87	£87	£87	£87	£87	£87
Consultant (hospital based)											
home visit	£0	£0	£0	£0	£0	£0	£0	£0	£0	£0	£0
surgery/hospital visit	£90	£90	£90	£90	£90	£90	£90	£90	£0	£90	£0
not face-to-face external interaction	£0	£0	£0	£0	£0	£0	£0	£0	£0	£0	£0
not face-to-face internal interaction	£0	£0	£0	£0	£0	£0	£0	£0	£0	£0	£0

Centre 2

Number of contacts

	Inherited metabolic disorders (IMD)						Hypothyroidism (CHT)	Sickle Cell Disorder (SCD)		Cystic Fibrosis (CF)	
			**isovaleric aciduria (IVA)**	**glutaric aciduria type 1 (GA1)**	**phenylketonuria (PKU)**	**homocystinuria (HCU)**					
**Personnel - Clinical path items**								NBS+: Affected	NBS+: Carrier	NBS+: Affected	NBS+: Carrier
CNS - Clinical nurse specialist											
home visit	1	1	1	1	1	1	1	1		2	
surgery/hospital visit	1	1	1	1	1	1	1	1		1	
not face-to-face external interaction	1	1	1	1	1	1	2	2		2	
not face-to-face internal interaction	3	3	3	3	3	3	3	2		3	
Health visitor/midwife											
home visit									1		1
surgery/hospital visit											
not face-to-face external interaction									2		2
not face-to-face internal interaction	1	1	1	1	1	1	1		1		3
Consumables											
Leaflet											
GP											
home visit											
surgery/hospital visit											
not face-to-face external interaction											
not face-to-face internal interaction	2	2	2	2	2	2	2	2	1	1	1
Consultant (hospital based)											
home visit											
surgery/hospital visit	1	1	1	1	1	1	1	1		1	
not face-to-face external interaction	1	1	1	1	1	1					
not face-to-face internal interaction	2	2	2	2	2	2	2	2		2	

Cost

	Inherited metabolic disorders (IMD)						Hypothyroidism (CHT)	Sickle Cell Disorder (SCD)		Cystic Fibrosis (CF)	
	**medium chain acyl CoA dehydrogenase deficiency (MCADD)**	**maple syrup urine disease (MSUD)**	**isovaleric aciduria (IVA)**	**glutaric aciduria type 1 (GA1)**	**phenylketonuria (PKU)**	**homocystinuria (HCU)**					
**Personnel - Clinical path items**								NBS+: Affected	NBS+: Carrier	NBS+: Affected	NBS+: Carrier
CNS - Clinical nurse specialist											
home visit	£104	£104	£104	£104	£104	£104	£104	£104	£0	£208	£0
surgery/hospital visit	£65	£65	£65	£65	£65	£65	£65	£65	£0	£65	£0
not face-to-face external interaction	£27	£27	£27	£27	£27	£27	£54	£54	£0	£54	£0
not face-to-face internal interaction	£80	£80	£80	£80	£80	£80	£80	£54	£0	£80	£0
Health visitor/midwife											
home visit	£0	£0	£0	£0	£0	£0	£0	£0	£104	£0	£104
surgery/hospital visit	£0	£0	£0	£0	£0	£0	£0	£0	£0	£0	£0
not face-to-face external interaction	£0	£0	£0	£0	£0	£0	£0	£0	£54	£0	£54
not face-to-face internal interaction	£27	£27	£27	£27	£27	£27	£27	£0	£27	£0	£80
Consumables											
Leaflet	£0	£0	£0	£0	£0	£0	£0	£0	£0	£0	£0
GP											
home visit	£0	£0	£0	£0	£0	£0	£0	£0	£0	£0	£0
surgery/hospital visit	£0	£0	£0	£0	£0	£0	£0	£0	£0	£0	£0
not face-to-face external interaction	£0	£0	£0	£0	£0	£0	£0	£0	£0	£0	£0
not face-to-face internal interaction	£175	£175	£175	£175	£175	£175	£175	£175	£87	£87	£87
Consultant (hospital based)											
home visit	£0	£0	£0	£0	£0	£0	£0	£0	£0	£0	£0
surgery/hospital visit	£90	£90	£90	£90	£90	£90	£90	£90	£0	£90	£0
not face-to-face external interaction	£37	£37	£37	£37	£37	£37	£0	£0	£0	£0	£0
not face-to-face internal interaction	£75	£75	£75	£75	£75	£75	£75	£75	£0	£75	£0

Centre 3

Number of contacts

	Inherited metabolic disorders (IMD)										
	medium chain acyl CoA dehydrogenase deficiency (MCADD)	maple syrup urine disease (MSUD)	isovaleric aciduria (IVA)	glutaric aciduria type 1 (GA1)	phenylketonuria (PKU)	homocystinuria (HCU)	Hypothyroidism (CHT)	Sickle Cell Disorder (SCD)		Cystic Fibrosis (CF)	
**Personnel - Clinical path items**								NBS+: Affected	NBS+: Carrier	NBS+: Affected	NBS+: Carrier
CNS - Clinical nurse specialist											
home visit	1	1	1	1	1	1	1	1	0	1	1
surgery/hospital visit	1	1	1	1	1	1	1	0	0	1	0
not face-to-face external interaction	2	2	2	2	2	2	1	3	0	3	0
not face-to-face internal interaction	3	3	3	3	3	3	1	2	0	2	2
Health visitor/midwife											
home visit	0	0	0	0	0	0	0	0	1	0	0
surgery/hospital visit	0	0	0	0	0	0	0	0	0	0	0
not face-to-face external interaction	0	0	0	0	0	0	0	0	2	0	0
not face-to-face internal interaction	0	0	0	0	0	0	1	1	3	0	1
Consumables											
Leaflet											
GP											
home visit	0	0	0	0	0	0	0	0	0	0	0
surgery/hospital visit	0	0	0	0	0	0	0	0	0	0	0
not face-to-face external interaction	0	0	0	0	0	0	0	0	0	0	0
not face-to-face internal interaction	0	0	0	0	0	0	1	1	1	1	1
Consultant (hospital based)											
home visit	0	0	0	0	0	0	0	0	0	0	0
surgery/hospital visit	1	1	1	1	1	1	1	1	0	1	0
not face-to-face external interaction	0	0	0	0	0	0	1	0	0	0	0
not face-to-face internal interaction	2	2	2	2	2	2	2	2	0	2	0

Cost

	Inherited metabolic disorders (IMD)										
	medium chain acyl CoA dehydrogenase deficiency (MCADD)	maple syrup urine disease (MSUD)	isovaleric aciduria (IVA)	glutaric aciduria type 1 (GA1)	phenylketonuria (PKU)	homocystinuria (HCU)	Hypothyroidism (CHT)	Sickle Cell Disorder (SCD)		Cystic Fibrosis (CF)	
**Personnel - Clinical path items**								NBS+: Affected	NBS+: Carrier	NBS+: Affected	NBS+: Carrier
CNS - Clinical nurse specialist											
home visit	£104	£104	£104	£104	£104	£104	£104	£104	£0	£104	£104
surgery/hospital visit	£65	£65	£65	£65	£65	£65	£65	£0	£0	£65	£0
not face-to-face external interaction	£54	£54	£54	£54	£54	£54	£27	£80	£0	£80	£0
not face-to-face internal interaction	£80	£80	£80	£80	£80	£80	£27	£54	£0	£54	£54
Health visitor/midwife											
home visit	£0	£0	£0	£0	£0	£0	£0	£0	£104	£0	£0
surgery/hospital visit	£0	£0	£0	£0	£0	£0	£0	£0	£0	£0	£0
not face-to-face external interaction	£0	£0	£0	£0	£0	£0	£0	£0	£54	£0	£0
not face-to-face internal interaction	£0	£0	£0	£0	£0	£0	£27	£27	£80	£0	£27
Consumables											
Leaflet	£0	£0	£0	£0	£0	£0	£0	£0	£0	£0	£0
GP											
home visit	£0	£0	£0	£0	£0	£0	£0	£0	£0	£0	£0
surgery/hospital visit	£0	£0	£0	£0	£0	£0	£0	£0	£0	£0	£0
not face-to-face external interaction	£0	£0	£0	£0	£0	£0	£0	£0	£0	£0	£0
not face-to-face internal interaction	£0	£0	£0	£0	£0	£0	£87	£87	£87	£87	£87
Consultant (hospital based)											
home visit	£0	£0	£0	£0	£0	£0	£0	£0	£0	£0	£0
surgery/hospital visit	£90	£90	£90	£90	£90	£90	£90	£90	£0	£90	£0
not face-to-face external interaction	£0	£0	£0	£0	£0	£0	£37	£0	£0	£0	£0
not face-to-face internal interaction	£75	£75	£75	£75	£75	£75	£75	£75	£0	£75	£0

Centre 4

Number of contacts

	Inherited metabolic disorders (IMD)										
	medium chain acyl CoA dehydrogenase deficiency (MCADD)	maple syrup urine disease (MSUD)	isovaleric aciduria (IVA)	glutaric aciduria type 1 (GA1)	phenylketonuria (PKU)	homocystinuria (HCU)	Hypothyroidism (CHT)	Sickle Cell Disorder (SCD)		Cystic Fibrosis (CF)	
**Personnel - Clinical path items**								NBS+: Affected	NBS+: Carrier	NBS+: Affected	NBS+: Carrier
CNS - Clinical nurse specialist											
home visit	1	1	1	1	1	1	1	1	1	0	1
surgery/hospital visit	1	1	1	1	1	1	1	1	0	0	0
not face-to-face external interaction	2	2	2	2	2	2	1	2	1	0	2
not face-to-face internal interaction	3	3	3	3	3	3	1	2	0	0	2
Health visitor/midwife											
home visit	0	0	0	0	0	0	0	0	0	0	0
surgery/hospital visit	0	0	0	0	0	0	0	0	0	0	0
not face-to-face external interaction	0	0	0	0	0	0	0	0	0	0	0
not face-to-face internal interaction	0	0	0	0	0	0	0	1	0	0	0
Consumables											
Leaflet											
GP											
home visit	0	0	0	0	0	0	0	0	0	0	0
surgery/hospital visit	0	0	0	0	0	0	0	0	0	0	0
not face-to-face external interaction	0	0	0	0	0	0	0	0	1	0	0
not face-to-face internal interaction	0	0	0	0	0	0	1	1	1	0	1
Consultant (hospital based)											
home visit	0	0	0	0	0	0	0	0	0	0	0
surgery/hospital visit	1	1	1	1	1	1	1	1	0	0	0
not face-to-face external interaction	0	0	0	0	0	0	2	0	0	0	0
not face-to-face internal interaction	2	2	2	2	2	2	3	1	1	0	0

Cost

	Inherited metabolic disorders (IMD)										
	medium chain acyl CoA dehydrogenase deficiency (MCADD)	maple syrup urine disease (MSUD)	isovaleric aciduria (IVA)	glutaric aciduria type 1 (GA1)	phenylketonuria (PKU)	homocystinuria (HCU)	Hypothyroidism (CHT)	Sickle Cell Disorder (SCD)		Cystic Fibrosis (CF)	
**Personnel - Clinical path items**								NBS+: Affected	NBS+: Carrier	NBS+: Affected	NBS+: Carrier
CNS - Clinical nurse specialist											
home visit	£104	£104	£104	£104	£104	£104	£104	£104	£104	£0	£104
surgery/hospital visit	£65	£65	£65	£65	£65	£65	£65	£65	£0	£0	£0
not face-to-face external interaction	£54	£54	£54	£54	£54	£54	£27	£54	£27	£0	£54
not face-to-face internal interaction	£80	£80	£80	£80	£80	£80	£27	£54	£0	£0	£54
Health visitor/midwife											
home visit	£0	£0	£0	£0	£0	£0	£0	£0	£0	£0	£0
surgery/hospital visit	£0	£0	£0	£0	£0	£0	£0	£0	£0	£0	£0
not face-to-face external interaction	£0	£0	£0	£0	£0	£0	£0	£0	£0	£0	£0
not face-to-face internal interaction	£0	£0	£0	£0	£0	£0	£0	£27	£0	£0	£0
Consumables											
Leaflet	£0	£0	£0	£0	£0	£0	£0	£0	£0	£0	£0
GP											
home visit	£0	£0	£0	£0	£0	£0	£0	£0	£0	£0	£0
surgery/hospital visit	£0	£0	£0	£0	£0	£0	£0	£0	£0	£0	£0
not face-to-face external interaction	£0	£0	£0	£0	£0	£0	£0	£0	£87	£0	£0
not face-to-face internal interaction	£0	£0	£0	£0	£0	£0	£87	£87	£87	£0	£87
Consultant (hospital based)											
home visit	£0	£0	£0	£0	£0	£0	£0	£0	£0	£0	£0
surgery/hospital visit	£90	£90	£90	£90	£90	£90	£90	£90	£0	£0	£0
not face-to-face external interaction	£0	£0	£0	£0	£0	£0	£75	£0	£0	£0	£0
not face-to-face internal interaction	£75	£75	£75	£75	£75	£75	£112	£37	£37	£0	£0

Centre 5

Number of contacts

	Inherited metabolic disorders (IMD)										
	**medium chain acyl CoA dehydrogenase deficiency (MCADD)**	**maple syrup urine disease (MSUD)**	**isovaleric aciduria (IVA)**	**glutaric aciduria type 1 (GA1)**	**phenylketonuria (PKU)**	**homocystinuria (HCU)**	**Hypothyroidism (CHT)**	**Sickle Cell Disorder (SCD)**		**Cystic Fibrosis (CF)**	
**Personnel - Clinical path items**								NBS+: Affected	NBS+: Carrier	NBS+: Affected	NBS+: Carrier
CNS - Clinical nurse specialist											
home visit	1	1	1	1	1	1	1	0	0	0	1
surgery/hospital visit	1	1	1	1	1	1	1	0	0	0	0
not face-to-face external interaction	2	2	2	2	2	2	1	0	0	0	1
not face-to-face internal interaction	3	3	3	3	3	3	3	0	0	0	7
Health visitor/midwife											
home visit	0	0	0	0	0	0	0	0	0	0	0
surgery/hospital visit	0	0	0	0	0	0	0	0	0	0	0
not face-to-face external interaction	0	0	0	0	0	0	0	0	0	0	0
not face-to-face internal interaction	0	0	0	0	0	0	0	0	0	0	2
Consumables											
Leaflet											
GP											
home visit	0	0	0	0	0	0	0	0	0	0	0
surgery/hospital visit	0	0	0	0	0	0	0	0	0	0	0
not face-to-face external interaction	0	0	0	0	0	0	0	0	0	0	0
not face-to-face internal interaction	0	0	0	0	0	0	2	0	0	0	2
Consultant (hospital based)											
home visit	0	0	0	0	0	0	0	0	0	0	0
surgery/hospital visit	1	1	1	1	1	1	1	0	0	0	0
not face-to-face external interaction	0	0	0	0	0	0	1	0	0	0	0
not face-to-face internal interaction	2	2	2	2	2	2	0	0	0	0	0

Cost

	Inherited metabolic disorders (IMD)						Hypothyroidism (CHT)	Sickle Cell Disorder (SCD)		Cystic Fibrosis (CF)	
	medium chain acyl CoA dehydrogenase deficiency (MCADD)	maple syrup urine disease (MSUD)	isovaleric aciduria (IVA)	glutaric aciduria type 1 (GA1)	phenylketonuria (PKU)	homocystinuria (HCU)					
**Personnel - Clinical path items**								NBS+: Affected	NBS+: Carrier	NBS+: Affected	NBS+: Carrier
CNS - Clinical nurse specialist											
home visit	£104	£104	£104	£104	£104	£104	£104	£0	£0	£0	£104
surgery/hospital visit	£65	£65	£65	£65	£65	£65	£65	£0	£0	£0	£0
not face-to-face external interaction	£54	£54	£54	£54	£54	£54	£27	£0	£0	£0	£27
not face-to-face internal interaction	£80	£80	£80	£80	£80	£80	£80	£0	£0	£0	£188
Health visitor/midwife											
home visit	£0	£0	£0	£0	£0	£0	£0	£0	£0	£0	£0
surgery/hospital visit	£0	£0	£0	£0	£0	£0	£0	£0	£0	£0	£0
not face-to-face external interaction	£0	£0	£0	£0	£0	£0	£0	£0	£0	£0	£0
not face-to-face internal interaction	£0	£0	£0	£0	£0	£0	£0	£0	£0	£0	£54
Consumables											
Leaflet	£0	£0	£0	£0	£0	£0	£0	£0	£0	£0	£0
GP											
home visit	£0	£0	£0	£0	£0	£0	£0	£0	£0	£0	£0
surgery/hospital visit	£0	£0	£0	£0	£0	£0	£0	£0	£0	£0	£0
not face-to-face external interaction	£0	£0	£0	£0	£0	£0	£0	£0	£0	£0	£0
not face-to-face internal interaction	£0	£0	£0	£0	£0	£0	£175	£0	£0	£0	£175
Consultant (hospital based)											
home visit	£0	£0	£0	£0	£0	£0	£0	£0	£0	£0	£0
surgery/hospital visit	£90	£90	£90	£90	£90	£90	£90	£0	£0	£0	£0
not face-to-face external interaction	£0	£0	£0	£0	£0	£0	£37	£0	£0	£0	£0
not face-to-face internal interaction	£75	£75	£75	£75	£75	£75	£0	£0	£0	£0	£0

Centre 6

Number of contacts

	Inherited metabolic disorders (IMD)										
	medium chain acyl CoA dehydrogenase deficiency (MCADD)	maple syrup urine disease (MSUD)	isovaleric aciduria (IVA)	glutaric aciduria type 1 (GA1)	phenylketonuria (PKU)	homocystinuria (HCU)	Hypothyroidism (CHT)	Sickle Cell Disorder (SCD)		Cystic Fibrosis (CF)	
**Personnel - Clinical path items**								NBS+: Affected	NBS+: Carrier	NBS+: Affected	NBS+: Carrier
CNS - Clinical nurse specialist											
home visit	1	1	1	1	1	1	1	1	1	1	1
surgery/hospital visit	1	1	1	1	1	1	1	1	0	1	0
not face-to-face external interaction	3	3	3	3	3	3	1	2	2	1	1
not face-to-face internal interaction	3	3	3	3	3	3	1	4	2	4	7
Health visitor/midwife											
home visit	0	0	0	0	0	0	0	0	0	0	0
surgery/hospital visit	0	0	0	0	0	0	0	0	0	0	0
not face-to-face external interaction	0	0	0	0	0	0	0	0	0	0	0
not face-to-face internal interaction	0	1	1	1	0	0	0	1	0	1	2
Consumables											
Leaflet											
GP											
home visit	0	0	0	0	0	0	0	0	0	0	0
surgery/hospital visit	0	0	0	0	0	0	0	0	0	0	0
not face-to-face external interaction	0	0	0	0	0	0	0	0	0	0	0
not face-to-face internal interaction	1	1	1	1	1	1	1	1	1	2	2
Consultant (hospital based)											
home visit	0	0	0	0	0	0	0	0	0	0	0
surgery/hospital visit	1	1	1	1	1	1	1	1	0	1	0
not face-to-face external interaction	0	0	0	0	0	0	1	0	0	0	0
not face-to-face internal interaction	1	1	0	0	1	1	2	1	0	1	0

Cost

	Inherited metabolic disorders (IMD)										
	**medium chain acyl CoA dehydrogenase deficiency (MCADD)**	**maple syrup urine disease (MSUD)**	**isovaleric aciduria (IVA)**	**glutaric aciduria type 1 (GA1)**	**phenylketonuria (PKU)**	**homocystinuria (HCU)**	**Hypothyroidism (CHT)**	**Sickle Cell Disorder (SCD)**		**Cystic Fibrosis (CF)**	
**Personnel - Clinical path items**								NBS+: Affected	NBS+: Carrier	NBS+: Affected	NBS+: Carrier
CNS - Clinical nurse specialist											
home visit	£104	£104	£104	£104	£104	£104	£104	£104	£104	£104	£104
surgery/hospital visit	£65	£65	£65	£65	£65	£65	£65	£65	£0	£65	£0
not face-to-face external interaction	£80	£80	£80	£80	£80	£80	£27	£54	£54	£27	£27
not face-to-face internal interaction	£80	£80	£80	£80	£80	£80	£27	£107	£54	£107	£188
Health visitor/midwife											
home visit	£0	£0	£0	£0	£0	£0	£0	£0	£0	£0	£0
surgery/hospital visit	£0	£0	£0	£0	£0	£0	£0	£0	£0	£0	£0
not face-to-face external interaction	£0	£0	£0	£0	£0	£0	£0	£0	£0	£0	£0
not face-to-face internal interaction	£0	£27	£27	£27	£0	£0	£0	£27	£0	£27	£54
Consumables											
Leaflet	£0	£0	£0	£0	£0	£0	£0	£0	£0	£0	£0
GP											
home visit	£0	£0	£0	£0	£0	£0	£0	£0	£0	£0	£0
surgery/hospital visit	£0	£0	£0	£0	£0	£0	£0	£0	£0	£0	£0
not face-to-face external interaction	£0	£0	£0	£0	£0	£0	£0	£0	£0	£0	£0
not face-to-face internal interaction	£87	£87	£87	£87	£87	£87	£87	£87	£87	£175	£175
Consultant (hospital based)											
home visit	£0	£0	£0	£0	£0	£0	£0	£0	£0	£0	£0
surgery/hospital visit	£90	£90	£90	£90	£90	£90	£90	£90	£0	£90	£0
not face-to-face external interaction	£0	£0	£0	£0	£0	£0	£37	£0	£0	£0	£0
not face-to-face internal interaction	£37	£37	£0	£0	£37	£37	£75	£37	£0	£37	£0

Centre 7

Number of contacts

	Inherited metabolic disorders (IMD)										
	medium chain acyl CoA dehydrogenase deficiency (MCADD)	maple syrup urine disease (MSUD)	isovaleric aciduria (IVA)	glutaric aciduria type 1 (GA1)	phenylketonuria (PKU)	homocystinuria (HCU)	Hypothyroidism (CHT)	Sickle Cell Disorder (SCD)		Cystic Fibrosis (CF)	
**Personnel - Clinical path items**								NBS+: Affected	NBS+: Carrier	NBS+: Affected	NBS+: Carrier
CNS - Clinical nurse specialist											
home visit	0	0	0	0	0	0	0	1	1	1	0
surgery/hospital visit	0	0	0	0	0	0	0	1	0	1	0
not face-to-face external interaction	0	0	0	0	0	0	0	2	2	1	0
not face-to-face internal interaction	0	0	0	0	0	0	0	4	2	3	0
Health visitor/midwife											
home visit	0	0	0	0	0	0	0	0	0	0	1
surgery/hospital visit	0	0	0	0	0	0	0	0	0	0	0
not face-to-face external interaction	0	0	0	0	0	0	0	0	0	0	2
not face-to-face internal interaction	0	0	0	0	0	0	0	1	0	0	2
Consumables											
Leaflet											
GP											
home visit	0	0	0	0	0	0	0	0	0	0	0
surgery/hospital visit	0	0	0	0	0	0	0	0	0	0	0
not face-to-face external interaction	0	0	0	0	0	0	0	0	0	0	0
not face-to-face internal interaction	0	0	0	0	0	0	0	1	1	1	1
Consultant (hospital based)											
home visit	0	0	0	0	0	0	0	0	0	0	0
surgery/hospital visit	0	0	0	0	0	0	0	1	0	1	0
not face-to-face external interaction	0	0	0	0	0	0	0	0	0	1	0
not face-to-face internal interaction	0	0	0	0	0	0	0	1	0	3	0

Cost

	Inherited metabolic disorders (IMD)										
	**medium chain acyl CoA dehydrogenase deficiency (MCADD)**	**maple syrup urine disease (MSUD)**	**isovaleric aciduria (IVA)**	**glutaric aciduria type 1 (GA1)**	**phenylketonuria (PKU)**	**homocystinuria (HCU)**	**Hypothyroidism (CHT)**	**Sickle Cell Disorder (SCD)**		**Cystic Fibrosis (CF)**	
**Personnel - Clinical path items**								NBS+: Affected	NBS+: Carrier	NBS+: Affected	NBS+: Carrier
CNS - Clinical nurse specialist											
home visit	£0	£0	£0	£0	£0	£0	£0	£104	£104	£104	£0
surgery/hospital visit	£0	£0	£0	£0	£0	£0	£0	£65	£0	£65	£0
not face-to-face external interaction	£0	£0	£0	£0	£0	£0	£0	£54	£54	£27	£0
not face-to-face internal interaction	£0	£0	£0	£0	£0	£0	£0	£107	£54	£80	£0
Health visitor/midwife											
home visit	£0	£0	£0	£0	£0	£0	£0	£0	£0	£0	£104
surgery/hospital visit	£0	£0	£0	£0	£0	£0	£0	£0	£0	£0	£0
not face-to-face external interaction	£0	£0	£0	£0	£0	£0	£0	£0	£0	£0	£54
not face-to-face internal interaction	£0	£0	£0	£0	£0	£0	£0	£27	£0	£0	£54
Consumables											
Leaflet	£0	£0	£0	£0	£0	£0	£0	£0	£0	£0	£0
GP											
home visit	£0	£0	£0	£0	£0	£0	£0	£0	£0	£0	£0
surgery/hospital visit	£0	£0	£0	£0	£0	£0	£0	£0	£0	£0	£0
not face-to-face external interaction	£0	£0	£0	£0	£0	£0	£0	£0	£0	£0	£0
not face-to-face internal interaction	£0	£0	£0	£0	£0	£0	£0	£87	£87	£87	£87
Consultant (hospital based)											
home visit	£0	£0	£0	£0	£0	£0	£0	£0	£0	£0	£0
surgery/hospital visit	£0	£0	£0	£0	£0	£0	£0	£90	£0	£90	£0
not face-to-face external interaction	£0	£0	£0	£0	£0	£0	£0	£0	£0	£37	£0
not face-to-face internal interaction	£0	£0	£0	£0	£0	£0	£0	£37	£0	£112	£0

Centre 8

Number of contacts

	Inherited metabolic disorders (IMD)										
	**medium chain acyl CoA dehydrogenase deficiency (MCADD)**	**maple syrup urine disease (MSUD)**	**isovaleric aciduria (IVA)**	**glutaric aciduria type 1 (GA1)**	**phenylketonuria (PKU)**	**homocystinuria (HCU)**	**Hypothyroidism (CHT)**	**Sickle Cell Disorder (SCD)**		**Cystic Fibrosis (CF)**	
**Personnel - Clinical path items**								NBS+: Affected	NBS+: Carrier	NBS+: Affected	NBS+: Carrier
CNS - Clinical nurse specialist											
home visit	1	1	1	1	1	1	1	1	0	1	1
surgery/hospital visit	1	1	1	1	1	1	1	1	0	1	0
not face-to-face external interaction	0	1	1	1	0	1	1	2	0	2	1
not face-to-face internal interaction	3	2	2	2	3	2	1	2	0	4	1
Health visitor/midwife											
home visit	1	0	0	0	1	0	0	0	1	1	1
surgery/hospital visit	0	0	0	0	0	0	0	0	0	0	0
not face-to-face external interaction	2	0	0	0	2	0	0	0	2	0	0
not face-to-face internal interaction	1	0	0	0	1	0	0	1	2	3	2
Consumables											
Leaflet											
GP											
home visit	0	0	0	0	0	0	0	0	0	0	0
surgery/hospital visit	0	0	0	0	0	0	0	0	0	0	0
not face-to-face external interaction	0	0	0	0	0	0	0	0	0	0	0
not face-to-face internal interaction	0	0	0	0	0	0	1	1	1	1	1
Consultant (hospital based)											
home visit	0	0	0	0	0	0	0	0	0	0	0
surgery/hospital visit	1	1	1	1	1	1	1	1	0	1	0
not face-to-face external interaction	0	1	1	1	0	1	1	0	0	0	0
not face-to-face internal interaction	0	1	1	1	0	1	3	1	0	1	0

Cost

	Inherited metabolic disorders (IMD)										
	medium chain acyl CoA dehydrogenase deficiency (MCADD)	maple syrup urine disease (MSUD)	isovaleric aciduria (IVA)	glutaric aciduria type 1 (GA1)	phenylketonuria (PKU)	homocystinuria (HCU)	Hypothyroidism (CHT)	Sickle Cell Disorder (SCD)		Cystic Fibrosis (CF)	
**Personnel - Clinical path items**								NBS+: Affected	NBS+: Carrier	NBS+: Affected	NBS+: Carrier
CNS - Clinical nurse specialist											
home visit	£104	£104	£104	£104	£104	£104	£104	£104	£0	£104	£104
surgery/hospital visit	£65	£65	£65	£65	£65	£65	£65	£65	£0	£65	£0
not face-to-face external interaction	£0	£27	£27	£27	£0	£27	£27	£54	£0	£54	£27
not face-to-face internal interaction	£80	£54	£54	£54	£80	£54	£27	£54	£0	£107	£27
Health visitor/midwife											
home visit	£104	£0	£0	£0	£104	£0	£0	£0	£104	£104	£104
surgery/hospital visit	£0	£0	£0	£0	£0	£0	£0	£0	£0	£0	£0
not face-to-face external interaction	£54	£0	£0	£0	£54	£0	£0	£0	£54	£0	£0
not face-to-face internal interaction	£27	£0	£0	£0	£27	£0	£0	£27	£54	£80	£54
Consumables											
Leaflet	£0	£0	£0	£0	£0	£0	£0	£0	£0	£0	£0
GP											
home visit	£0	£0	£0	£0	£0	£0	£0	£0	£0	£0	£0
surgery/hospital visit	£0	£0	£0	£0	£0	£0	£0	£0	£0	£0	£0
not face-to-face external interaction	£0	£0	£0	£0	£0	£0	£0	£0	£0	£0	£0
not face-to-face internal interaction	£0	£0	£0	£0	£0	£0	£87	£87	£87	£87	£87
Consultant (hospital based)											
home visit	£0	£0	£0	£0	£0	£0	£0	£0	£0	£0	£0
surgery/hospital visit	£90	£90	£90	£90	£90	£90	£90	£90	£0	£90	£0
not face-to-face external interaction	£0	£37	£37	£37	£0	£37	£37	£0	£0	£0	£0
not face-to-face internal interaction	£0	£37	£37	£37	£0	£37	£112	£37	£0	£37	£0

Centre 9

Number of contacts

	Inherited metabolic disorders (IMD)						Hypothyroidism (CHT)	Sickle Cell Disorder (SCD)		Cystic Fibrosis (CF)	
	medium chain acyl CoA dehydrogenase deficiency (MCADD)	maple syrup urine disease (MSUD)	isovaleric aciduria (IVA)	glutaric aciduria type 1 (GA1)	phenylketonuria (PKU)	homocystinuria (HCU)					
**Personnel - Clinical path items**								NBS+: Affected	NBS+: Carrier	NBS+: Affected	NBS+: Carrier
CNS - Clinical nurse specialist											
home visit	1	1	1	1	1	1	0	1	0	0	0
surgery/hospital visit	1	1	1	1	1	1	1	1	0	1	0
not face-to-face external interaction	1	1	1	1	1	1	0	2	0	0	0
not face-to-face internal interaction	1	1	1	1	1	1	0	1	0	2	0
Health visitor/midwife											
home visit	0	0	0	0	0	0	1	0	1	1	1
surgery/hospital visit	0	0	0	0	0	0	0	0	0	0	0
not face-to-face external interaction	1	1	1	1	1	1	2	0	2	2	2
not face-to-face internal interaction	1	1	1	1	1	1	1	1	1	2	1
Consumables											
Leaflet											
GP											
home visit	0	0	0	0	0	0	0	0	0	0	0
surgery/hospital visit	0	0	0	0	0	0	0	0	0	0	0
not face-to-face external interaction	0	0	0	0	0	0	0	0	0	0	0
not face-to-face internal interaction	1	1	1	1	1	1	1	2	1	1	1
Consultant (hospital based)											
home visit	0	0	0	0	0	0	0	0	0	0	0
surgery/hospital visit	1	1	1	1	1	1	1	1	0	1	0
not face-to-face external interaction	0	0	0	0	0	0	0	0	0	0	0
not face-to-face internal interaction	1	1	1	1	1	1	1	0	0	0	0

Cost

	Inherited metabolic disorders (IMD)										
	**medium chain acyl CoA dehydrogenase deficiency (MCADD)**	**maple syrup urine disease (MSUD)**	**isovaleric aciduria (IVA)**	**glutaric aciduria type 1 (GA1)**	**phenylketonuria (PKU)**	**homocystinuria (HCU)**	**Hypothyroidism (CHT)**	**Sickle Cell Disorder (SCD)**		**Cystic Fibrosis (CF)**	
**Personnel - Clinical path items**								NBS+: Affected	NBS+: Carrier	NBS+: Affected	NBS+: Carrier
CNS - Clinical nurse specialist											
home visit	£104	£104	£104	£104	£104	£104	£0	£104	£0	£0	£0
surgery/hospital visit	£65	£65	£65	£65	£65	£65	£65	£65	£0	£65	£0
not face-to-face external interaction	£27	£27	£27	£27	£27	£27	£0	£54	£0	£0	£0
not face-to-face internal interaction	£27	£27	£27	£27	£27	£27	£0	£27	£0	£54	£0
Health visitor/midwife											
home visit	£0	£0	£0	£0	£0	£0	£104	£0	£104	£104	£104
surgery/hospital visit	£0	£0	£0	£0	£0	£0	£0	£0	£0	£0	£0
not face-to-face external interaction	£27	£27	£27	£27	£27	£27	£54	£0	£54	£54	£54
not face-to-face internal interaction	£27	£27	£27	£27	£27	£27	£27	£27	£27	£54	£27
Consumables											
Leaflet	£0	£0	£0	£0	£0	£0	£0	£0	£0	£0	£0
GP											
home visit	£0	£0	£0	£0	£0	£0	£0	£0	£0	£0	£0
surgery/hospital visit	£0	£0	£0	£0	£0	£0	£0	£0	£0	£0	£0
not face-to-face external interaction	£0	£0	£0	£0	£0	£0	£0	£0	£0	£0	£0
not face-to-face internal interaction	£87	£87	£87	£87	£87	£87	£87	£175	£87	£87	£87
Consultant (hospital based)											
home visit	£0	£0	£0	£0	£0	£0	£0	£0	£0	£0	£0
surgery/hospital visit	£90	£90	£90	£90	£90	£90	£90	£90	£0	£90	£0
not face-to-face external interaction	£0	£0	£0	£0	£0	£0	£0	£0	£0	£0	£0
not face-to-face internal interaction	£37	£37	£37	£37	£37	£37	£37	£0	£0	£0	£0

Centre 10

Number of contacts

	Inherited metabolic disorders (IMD)						Hypothyroidism (CHT)	Sickle Cell Disorder (SCD)		Cystic Fibrosis (CF)	
	medium chain acyl CoA dehydrogenase deficiency (MCADD)	maple syrup urine disease (MSUD)	isovaleric aciduria (IVA)	glutaric aciduria type 1 (GA1)	phenylketonuria (PKU)	homocystinuria (HCU)					
**Personnel - Clinical path items**								NBS+: Affected	NBS+: Carrier	NBS+: Affected	NBS+: Carrier
CNS - Clinical nurse specialist											
home visit	1	1	1	1	1	1	0	1	1	1	1
surgery/hospital visit	1	1	1	1	1	1	1	1	0	1	0
not face-to-face external interaction	0	1	1	1	0	1	0	2	2	2	1
not face-to-face internal interaction	3	2	2	2	3	2	1	4	2	4	0
Health visitor/midwife											
home visit	1	0	0	0	1	0	1	0	0	1	0
surgery/hospital visit	0	0	0	0	0	0	0	0	0	0	0
not face-to-face external interaction	2	0	0	0	2	0	2	1	0	0	1
not face-to-face internal interaction	1	0	0	0	1	0	1	1	0	2	2
Consumables											
Leaflet											
GP											
home visit	0	0	0	0	0	0	0	0	0	0	0
surgery/hospital visit	0	0	0	0	0	0	0	0	0	0	0
not face-to-face external interaction	0	0	0	0	0	0	0	0	0	0	0
not face-to-face internal interaction	0	0	0	0	0	0	0	1	1	0	0
Consultant (hospital based)											
home visit	0	0	0	0	0	0	0	0	0	0	0
surgery/hospital visit	1	1	1	1	1	1	1	1	0	1	0
not face-to-face external interaction	0	1	1	1	0	1	0	0	0	0	0
not face-to-face internal interaction	0	1	1	1	0	1	1	1	0	1	0

Cost

	Inherited metabolic disorders (IMD)										
	medium chain acyl CoA dehydrogenase deficiency (MCADD)	maple syrup urine disease (MSUD)	isovaleric aciduria (IVA)	glutaric aciduria type 1 (GA1)	phenylketonuria (PKU)	homocystinuria (HCU)	Hypothyroidism (CHT)	Sickle Cell Disorder (SCD)		Cystic Fibrosis (CF)	
**Personnel - Clinical path items**								NBS+: Affected	NBS+: Carrier	NBS+: Affected	NBS+: Carrier
CNS - Clinical nurse specialist											
home visit	£104	£104	£104	£104	£104	£104	£0	£104	£104	£104	£104
surgery/hospital visit	£65	£65	£65	£65	£65	£65	£65	£65	£0	£65	£0
not face-to-face external interaction	£0	£27	£27	£27	£0	£27	£0	£54	£54	£54	£27
not face-to-face internal interaction	£80	£54	£54	£54	£80	£54	£27	£107	£54	£107	£0
Health visitor/midwife											
home visit	£104	£0	£0	£0	£104	£0	£104	£0	£0	£104	£0
surgery/hospital visit	£0	£0	£0	£0	£0	£0	£0	£0	£0	£0	£0
not face-to-face external interaction	£54	£0	£0	£0	£54	£0	£54	£27	£0	£0	£27
not face-to-face internal interaction	£27	£0	£0	£0	£27	£0	£27	£27	£0	£54	£54
Consumables											
Leaflet	£0	£0	£0	£0	£0	£0	£0	£0	£0	£0	£0
GP											
home visit	£0	£0	£0	£0	£0	£0	£0	£0	£0	£0	£0
surgery/hospital visit	£0	£0	£0	£0	£0	£0	£0	£0	£0	£0	£0
not face-to-face external interaction	£0	£0	£0	£0	£0	£0	£0	£0	£0	£0	£0
not face-to-face internal interaction	£0	£0	£0	£0	£0	£0	£0	£87	£87	£0	£0
Consultant (hospital based)											
home visit	£0	£0	£0	£0	£0	£0	£0	£0	£0	£0	£0
surgery/hospital visit	£90	£90	£90	£90	£90	£90	£90	£90	£0	£90	£0
not face-to-face external interaction	£0	£37	£37	£37	£0	£37	£0	£0	£0	£0	£0
not face-to-face internal interaction	£0	£37	£37	£37	£0	£37	£37	£37	£0	£37	£0

Centre 11

Number of contacts

	Inherited metabolic disorders (IMD)										
	medium chain acyl CoA dehydrogenase deficiency (MCADD)	maple syrup urine disease (MSUD)	isovaleric aciduria (IVA)	glutaric aciduria type 1 (GA1)	phenylketonuria (PKU)	homocystinuria (HCU)	Hypothyroidism (CHT)	Sickle Cell Disorder (SCD)		Cystic Fibrosis (CF)	
**Personnel - Clinical path items**								NBS+: Affected	NBS+: Carrier	NBS+: Affected	NBS+: Carrier
CNS - Clinical nurse specialist											
home visit	1	1	1	1	1	1	1	1	0	0	1
surgery/hospital visit	1	1	1	1	1	1	1	1	0	1	0
not face-to-face external interaction	0	0	0	0	0	0	1	2	2	2	2
not face-to-face internal interaction	3	3	3	3	3	3	2	4	2	5	3
Health visitor/midwife											
home visit	0	0	0	0	0	0	0	0	1	2	0
surgery/hospital visit	0	0	0	0	0	0	0	0		0	0
not face-to-face external interaction	0	0	0	0	0	0	0	0	2	0	0
not face-to-face internal interaction	0	0	0	0	0	0	0	0	1	3	0
Consumables											
Leaflet											
GP											
home visit	0	0	0	0	0	0	0	0	0	0	0
surgery/hospital visit	0	0	0	0	0	0	0	0	0	0	0
not face-to-face external interaction	0	0	0	0	0	0	1	0	0	0	0
not face-to-face internal interaction	1	1	1	1	1	1	1	1	0	1	0
Consultant (hospital based)											
home visit	0	0	0	0	0	0	0	0	0	0	0
surgery/hospital visit	1	1	1	1	1	1	1	1	0	1	0
not face-to-face external interaction	1	1	1	1	1	1	0	0	0	0	0
not face-to-face internal interaction	4	4	4	4	4	4	2	2	0	2	2

Cost

	Inherited metabolic disorders (IMD)						Hypothyroidism (CHT)	Sickle Cell Disorder (SCD)		Cystic Fibrosis (CF)	
	medium chain acyl CoA dehydrogenase deficiency (MCADD)	maple syrup urine disease (MSUD)	isovaleric aciduria (IVA)	glutaric aciduria type 1 (GA1)	phenylketonuria (PKU)	homocystinuria (HCU)					
**Personnel - Clinical path items**								NBS+: Affected	NBS+: Carrier	NBS+: Affected	NBS+: Carrier
CNS - Clinical nurse specialist											
home visit	£104	£104	£104	£104	£104	£104	£104	£104	£0	£0	£104
surgery/hospital visit	£65	£65	£65	£65	£65	£65	£65	£65	£0	£65	£0
not face-to-face external interaction	£0	£0	£0	£0	£0	£0	£27	£54	£54	£54	£54
not face-to-face internal interaction	£80	£80	£80	£80	£80	£80	£54	£107	£54	£134	£80
Health visitor/midwife											
home visit	£0	£0	£0	£0	£0	£0	£0	£0	£104	£208	£0
surgery/hospital visit	£0	£0	£0	£0	£0	£0	£0	£0	£0	£0	£0
not face-to-face external interaction	£0	£0	£0	£0	£0	£0	£0	£0	£54	£0	£0
not face-to-face internal interaction	£0	£0	£0	£0	£0	£0	£0	£0	£27	£80	£0
Consumables											
Leaflet	£0	£0	£0	£0	£0	£0	£0	£0	£0	£0	£0
GP											
home visit	£0	£0	£0	£0	£0	£0	£0	£0	£0	£0	£0
surgery/hospital visit	£0	£0	£0	£0	£0	£0	£0	£0	£0	£0	£0
not face-to-face external interaction	£0	£0	£0	£0	£0	£0	£87	£0	£0	£0	£0
not face-to-face internal interaction	£87	£87	£87	£87	£87	£87	£87	£87	£0	£87	£0
Consultant (hospital based)											
home visit	£0	£0	£0	£0	£0	£0	£0	£0	£0	£0	£0
surgery/hospital visit	£90	£90	£90	£90	£90	£90	£90	£90	£0	£90	£0
not face-to-face external interaction	£37	£37	£37	£37	£37	£37	£0	£0	£0	£0	£0
not face-to-face internal interaction	£150	£150	£150	£150	£150	£150	£75	£75	£0	£75	£75

Centre 12

Number of contacts

	Inherited metabolic disorders (IMD)										
	**medium chain acyl CoA dehydrogenase deficiency (MCADD)**	**maple syrup urine disease (MSUD)**	**isovaleric aciduria (IVA)**	**glutaric aciduria type 1 (GA1)**	**phenylketonuria (PKU)**	**homocystinuria (HCU)**	**Hypothyroidism (CHT)**	**Sickle Cell Disorder (SCD)**		**Cystic Fibrosis (CF)**	
**Personnel - Clinical path items**								NBS+: Affected	NBS+: Carrier	NBS+: Affected	NBS+: Carrier
CNS - Clinical nurse specialist											
home visit	1	1	1	1	1	1	1	1	1	1	1
surgery/hospital visit	1	1	1	1	1	1	1	1	0	1	0
not face-to-face external interaction	2	2	2	2	2	2	2	2	2	2	3
not face-to-face internal interaction	4	4	4	4	4	4	5	3	2	6	2
Health visitor/midwife											
home visit	0	0	0	0	0	0	0	0	0	0	0
surgery/hospital visit	0	0	0	0	0	0	0	0	0	0	0
not face-to-face external interaction	0	0	0	0	0	0	0	0	0	0	0
not face-to-face internal interaction	1	1	1	1	1	1	1	0	0	1	0
Consumables											
Leaflet											
GP											
home visit	0	0	0	0	0	0	0	0	0	0	0
surgery/hospital visit	0	0	0	0	0	0	0	0	0	0	0
not face-to-face external interaction	0	0	0	0	0	0	0	0	0	0	0
not face-to-face internal interaction	2	2	2	2	2	2	2	1	1	2	1
Consultant (hospital based)											
home visit	0	0	0	0	0	0	0	0	0	0	0
surgery/hospital visit	1	1	1	1	1	1	1	1	0	1	0
not face-to-face external interaction	0	0	0	0	0	0	0	0	0	0	0
not face-to-face internal interaction	2	2	2	2	2	2	3	2	0	1	0

Cost

	Inherited metabolic disorders (IMD)										
	medium chain acyl CoA dehydrogenase deficiency (MCADD)	maple syrup urine disease (MSUD)	isovaleric aciduria (IVA)	glutaric aciduria type 1 (GA1)	phenylketonuria (PKU)	homocystinuria (HCU)	Hypothyroidism (CHT)	Sickle Cell Disorder (SCD)		Cystic Fibrosis (CF)	
**Personnel - Clinical path items**								NBS+: Affected	NBS+: Carrier	NBS+: Affected	NBS+: Carrier
CNS - Clinical nurse specialist											
home visit	£104	£104	£104	£104	£104	£104	£104	£104	£104	£104	£104
surgery/hospital visit	£65	£65	£65	£65	£65	£65	£65	£65	£0	£65	£0
not face-to-face external interaction	£54	£54	£54	£54	£54	£54	£54	£54	£54	£54	£80
not face-to-face internal interaction	£107	£107	£107	£107	£107	£107	£134	£80	£54	£161	£54
Health visitor/midwife											
home visit	£0	£0	£0	£0	£0	£0	£0	£0	£0	£0	£0
surgery/hospital visit	£0	£0	£0	£0	£0	£0	£0	£0	£0	£0	£0
not face-to-face external interaction	£0	£0	£0	£0	£0	£0	£0	£0	£0	£0	£0
not face-to-face internal interaction	£27	£27	£27	£27	£27	£27	£27	£0	£0	£27	£0
Consumables											
Leaflet	£0	£0	£0	£0	£0	£0	£0	£0	£0	£0	£0
GP											
home visit	£0	£0	£0	£0	£0	£0	£0	£0	£0	£0	£0
surgery/hospital visit	£0	£0	£0	£0	£0	£0	£0	£0	£0	£0	£0
not face-to-face external interaction	£0	£0	£0	£0	£0	£0	£0	£0	£0	£0	£0
not face-to-face internal interaction	£175	£175	£175	£175	£175	£175	£175	£87	£87	£175	£87
Consultant (hospital based)											
home visit	£0	£0	£0	£0	£0	£0	£0	£0	£0	£0	£0
surgery/hospital visit	£90	£90	£90	£90	£90	£90	£90	£90	£0	£90	£0
not face-to-face external interaction	£0	£0	£0	£0	£0	£0	£0	£0	£0	£0	£0
not face-to-face internal interaction	£75	£75	£75	£75	£75	£75	£112	£75	£0	£37	£0

Centre 13

Number of contacts

	Inherited metabolic disorders (IMD)										
	medium chain acyl CoA dehydrogenase deficiency (MCADD)	maple syrup urine disease (MSUD)	isovaleric aciduria (IVA)	glutaric aciduria type 1 (GA1)	phenylketonuria (PKU)	homocystinuria (HCU)	Hypothyroidism (CHT)	Sickle Cell Disorder (SCD)		Cystic Fibrosis (CF)	
**Personnel - Clinical path items**								NBS+: Affected	NBS+: Carrier	NBS+: Affected	NBS+: Carrier
CNS - Clinical nurse specialist											
home visit	1	1	1	1	1	1	1	1	1	1	1
surgery/hospital visit	1	1	1	1	1	1	1	1	0	1	0
not face-to-face external interaction	1	2	2	2	1	2	1	1	2	2	2
not face-to-face internal interaction	1	3	3	3	1	3	3	0	2	4	4
Health visitor/midwife											
home visit	0	0	0	0	0	0	0	0	0	0	
surgery/hospital visit	0	0	0	0	0	0	0	0	0	0	
not face-to-face external interaction	0	0	0	0	0	0	0	0	0	0	
not face-to-face internal interaction	0	0	0	0	0	0	1	1	2	2	1
Consumables											
Leaflet											
GP											
home visit	0	0	0	0	0	0	0	0	0	0	
surgery/hospital visit	0	0	0	0	0	0	0	0	0	0	
not face-to-face external interaction	0	0	0	0	0	0	0	0	0	0	
not face-to-face internal interaction	0	0	0	0	0	0	2	1	1	3	2
Consultant (hospital based)											
home visit	0	0	0	0	0	0	0	0	0	0	0
surgery/hospital visit	1	1	1	1	1	1	1	1	0	1	0
not face-to-face external interaction	1	0	0	0	1	0	1	0	0	0	0
not face-to-face internal interaction	1	2	2	2	1	2	4	6	0	4	0

Cost

	Inherited metabolic disorders (IMD)										
	**medium chain acyl CoA dehydrogenase deficiency (MCADD)**	**maple syrup urine disease (MSUD)**	**isovaleric aciduria (IVA)**	**glutaric aciduria type 1 (GA1)**	**phenylketonuria (PKU)**	**homocystinuria (HCU)**	**Hypothyroidism (CHT)**	**Sickle Cell Disorder (SCD)**		**Cystic Fibrosis (CF)**	
**Personnel - Clinical path items**								NBS+: Affected	NBS+: Carrier	NBS+: Affected	NBS+: Carrier
CNS - Clinical nurse specialist											
home visit	£104	£104	£104	£104	£104	£104	£104	£104	£104	£104	£104
surgery/hospital visit	£65	£65	£65	£65	£65	£65	£65	£65	£0	£65	£0
not face-to-face external interaction	£27	£54	£54	£54	£27	£54	£27	£27	£54	£54	£54
not face-to-face internal interaction	£27	£80	£80	£80	£27	£80	£80	£0	£54	£107	£107
Health visitor/midwife											
home visit	£0	£0	£0	£0	£0	£0	£0	£0	£0	£0	£0
surgery/hospital visit	£0	£0	£0	£0	£0	£0	£0	£0	£0	£0	£0
not face-to-face external interaction	£0	£0	£0	£0	£0	£0	£0	£0	£0	£0	£0
not face-to-face internal interaction	£0	£0	£0	£0	£0	£0	£27	£27	£54	£54	£27
Consumables											
Leaflet	£0	£0	£0	£0	£0	£0	£0	£0	£0	£0	£0
GP											
home visit	£0	£0	£0	£0	£0	£0	£0	£0	£0	£0	£0
surgery/hospital visit	£0	£0	£0	£0	£0	£0	£0	£0	£0	£0	£0
not face-to-face external interaction	£0	£0	£0	£0	£0	£0	£0	£0	£0	£0	£0
not face-to-face internal interaction	£0	£0	£0	£0	£0	£0	£175	£87	£87	£262	£175
Consultant (hospital based)											
home visit	£0	£0	£0	£0	£0	£0	£0	£0	£0	£0	£0
surgery/hospital visit	£90	£90	£90	£90	£90	£90	£90	£90	£0	£90	£0
not face-to-face external interaction	£37	£0	£0	£0	£37	£0	£37	£0	£0	£0	£0
not face-to-face internal interaction	£37	£75	£75	£75	£37	£75	£150	£224	£0	£150	£0

Centre 14

Number of contacts

	Inherited metabolic disorders (IMD)										
	**medium chain acyl CoA dehydrogenase deficiency (MCADD)**	**maple syrup urine disease (MSUD)**	**isovaleric aciduria (IVA)**	**glutaric aciduria type 1 (GA1)**	**phenylketonuria (PKU)**	**homocystinuria (HCU)**	**Hypothyroidism (CHT)**	**Sickle Cell Disorder (SCD)**		**Cystic Fibrosis (CF)**	
**Personnel - Clinical path items**								NBS+: Affected	NBS+: Carrier	NBS+: Affected	NBS+: Carrier
CNS - Clinical nurse specialist											
home visit	1	1	1	1	1	1	1	1	1	1	0
surgery/hospital visit	1	1	1	1	1	1	1	1	0	1	0
not face-to-face external interaction	2	2	2	2	2	2	1	2	3	2	2
not face-to-face internal interaction	4	4	4	4	4	4	0	4	4	5	3
Health visitor/midwife											
home visit	0	0	0	0	0	0	0	0	0	0	1
surgery/hospital visit	0	0	0	0	0	0	0	0	0	0	0
not face-to-face external interaction	0	0	0	0	0	0	0	0	0	0	0
not face-to-face internal interaction	1	1	1	1	1	1	0	1	1	1	1
Consumables											
Leaflet											
GP											
home visit	0	0	0	0	0	0	0	0	0	0	0
surgery/hospital visit	0	0	0	0	0	0	0	0	0	0	0
not face-to-face external interaction	0	0	0	0	0	0	0	0	0	0	0
not face-to-face internal interaction	1	1	1	1	1	1	1	1	1	1	0
Consultant (hospital based)											
home visit	0	0	0	0	0	0	0	0	0	0	0
surgery/hospital visit	1	1	1	1	1	1	1	1	0	1	0
not face-to-face external interaction	0	0	0	0	0	0	1	0	0	0	0
not face-to-face internal interaction	3	3	3	3	3	3	4	3	0	0	0

Cost

	Inherited metabolic disorders (IMD)										
	**medium chain acyl CoA dehydrogenase deficiency (MCADD)**	**maple syrup urine disease (MSUD)**	**isovaleric aciduria (IVA)**	**glutaric aciduria type 1 (GA1)**	**phenylketonuria (PKU)**	**homocystinuria (HCU)**	**Hypothyroidism (CHT)**	**Sickle Cell Disorder (SCD)**		**Cystic Fibrosis (CF)**	
**Personnel - Clinical path items**								NBS+: Affected	NBS+: Carrier	NBS+: Affected	NBS+: Carrier
CNS - Clinical nurse specialist											
home visit	£104	£104	£104	£104	£104	£104	£104	£104	£104	£104	£0
surgery/hospital visit	£65	£65	£65	£65	£65	£65	£65	£65	£0	£65	£0
not face-to-face external interaction	£54	£54	£54	£54	£54	£54	£27	£54	£80	£54	£54
not face-to-face internal interaction	£107	£107	£107	£107	£107	£107	£0	£107	£107	£134	£80
Health visitor/midwife											
home visit	£0	£0	£0	£0	£0	£0	£0	£0	£0	£0	£104
surgery/hospital visit	£0	£0	£0	£0	£0	£0	£0	£0	£0	£0	£0
not face-to-face external interaction	£0	£0	£0	£0	£0	£0	£0	£0	£0	£0	£0
not face-to-face internal interaction	£27	£27	£27	£27	£27	£27	£0	£27	£27	£27	£27
Consumables											
Leaflet	£0	£0	£0	£0	£0	£0	£0	£0	£0	£0	£0
GP											
home visit	£0	£0	£0	£0	£0	£0	£0	£0	£0	£0	£0
surgery/hospital visit	£0	£0	£0	£0	£0	£0	£0	£0	£0	£0	£0
not face-to-face external interaction	£0	£0	£0	£0	£0	£0	£0	£0	£0	£0	£0
not face-to-face internal interaction	£87	£87	£87	£87	£87	£87	£87	£87	£87	£87	£0
Consultant (hospital based)											
home visit	£0	£0	£0	£0	£0	£0	£0	£0	£0	£0	£0
surgery/hospital visit	£90	£90	£90	£90	£90	£90	£90	£90	£0	£90	£0
not face-to-face external interaction	£0	£0	£0	£0	£0	£0	£37	£0	£0	£0	£0
not face-to-face internal interaction	£112	£112	£112	£112	£112	£112	£150	£112	£0	£0	£0
