# Supplementary material for: Delivering Positive Newborn Screening Results: Cost Analysis of Existing Practice versus Innovative, Co-Designed Strategies from the ReSPoND Study
Source: Int J Neonatal Screen. 2022 Mar 14;8(1):19. doi: 10.3390/ijns8010019 (PMC8951105; doi:10.3390/ijns8010019)
Supplement: Supplementary file 1 [file IJNS-08-00019-s001.zip › Table S4 Sensitivity analysis time - RESPOND (blinded)_rev.pdf]

Table S4. Sensitivity analysis: Average cost (95% Confidence Intervals) (£)

	MCADD	MSUD	IVA	GA1	PKU	HCU	CHT	SCD - affected	SCD - carrier	Cf - affected	Cf - carrier
**Basecase**											
SP	437.02 (372.34 to 504.98)	444.47 (375.61 to 516.82)	440.73 (372.34 to 513.85)	466.87 (383.31 to 531.79)	470.49 (383.95 to 533.55)	462.56 (379.15 to 527.23)	500.64 (446.15 to 556.51)	564.14 (504.36 to 609.83)	237.12 (190.58 to 279.04)	628.51 (582.22 to 687.9)	265.35 (196.23 to 331.83)
IP - home visit	542.68 (484.71 to 603.17)	549.97 (488.83 to 614.19)	546.23 (486.19 to 609.63)	546.23 (486.19 to 609.63)	550.19 (487.85 to 614.93)	547.28 (486.6 to 610.31)	574.71 (523.64 to 634.67)	565.97 (540.73 to 590.04)	312.84 (292.93 to 328.97)	646.39 (597.88 to 719.74)	355.36 (302.58 to 417.4)
IP - teleconsultation	473.91 (413.37 to 530.01)	490.07 (422.16 to 552.52)	483.65 (419.7 to 542.68)	475.6 (413.26 to 540.43)	474.2 (410.95 to 539.18)	479 (415.65 to 537.91)	497.67 (446.59 to 557.62)	488.92 (463.69 to 513)	235.79 (215.88 to 251.92)	552.03 (502.98 to 623.42)	268.26 (213.87 to 331.19)
**Home visit time -25%**											
SP	432.3 (367.53 to 500.78)	444.47 (375.61 to 516.82)	440.73 (372.34 to 513.85)	461.67 (382.32 to 526.25)	460.1 (378.49 to 524.31)	457.36 (378.47 to 522.37)	492.65 (437.31 to 550.98)	538.17 (482.33 to 580.8)	217.64 (176.89 to 253.08)	593.88 (552.16 to 650.55)	249.37 (184.65 to 316.44)
IP - home visit	514.35 (456.28 to 575.07)	524 (462.87 to 588.23)	520.26 (460.22 to 583.66)	520.26 (460.22 to 583.66)	521.63 (458.66 to 586.8)	521.31 (460.63 to 584.34)	548.75 (497.67 to 608.7)	540 (514.77 to 564.07)	286.87 (266.96 to 303)	611.76 (564.91 to 683.7)	325.4 (274.43 to 387.49)
IP - teleconsultation	471.55 (410.8 to 528.1)	490.07 (422.16 to 552.52)	483.65 (419.7 to 542.68)	475.6 (413.26 to 540.43)	471.6 (407.81 to 537.6)	476.4 (414.32 to 536.8)	497.67 (446.59 to 557.62)	488.92 (463.69 to 513)	235.79 (215.88 to 251.92)	547.7 (499.77 to 618.33)	266.26 (213.19 to 329.35)
**Home visit time +25%**											
SP	441.74 (374.73 to 511.24)	444.47 (375.61 to 516.82)	440.73 (372.34 to 513.85)	472.06 (384.11 to 536.43)	480.88 (388.57 to 546.37)	467.75 (379.45 to 533.05)	508.63 (453.47 to 563.11)	590.1 (524.18 to 640.19)	256.59 (204.75 to 305.95)	663.13 (609.8 to 727.98)	281.33 (207.21 to 348.39)
IP - home visit	571.01 (513.31 to 631.69)	575.93 (514.8 to 640.16)	572.19 (512.16 to 635.6)	572.19 (512.16 to 635.6)	578.76 (516.18 to 643.06)	573.25 (512.57 to 636.28)	600.68 (549.6 to 660.64)	591.93 (566.7 to 616.01)	338.81 (318.9 to 354.94)	681.01 (630.75 to 756.63)	385.32 (330.8 to 447.6)
IP - teleconsultation	476.27 (416.09 to 531.63)	490.07 (422.16 to 552.52)	483.65 (419.7 to 542.68)	475.6 (413.26 to 540.43)	476.8 (413.45 to 541.4)	481.59 (416.46 to 539.34)	497.67 (446.59 to 557.62)	488.92 (463.69 to 513)	235.79 (215.88 to 251.92)	556.36 (505.06 to 629.65)	270.26 (214.58 to 332.78)
**Hospital visit time -25%**											
SP	398.32 (333.63 to 466.28)	405.76 (336.91 to 478.12)	402.02 (333.63 to 475.15)	428.16 (344.61 to 493.08)	431.79 (345.25 to 494.85)	423.85 (340.45 to 488.53)	461.94 (407.45 to 517.81)	526.78 (466.96 to 571.95)	237.12 (190.58 to 279.04)	589.8 (543.52 to 649.2)	265.35 (196.23 to 331.83)
IP - home visit	503.98 (446 to 564.47)	511.26 (450.13 to 575.49)	507.52 (447.49 to 570.92)	507.52 (447.49 to 570.92)	511.49 (449.15 to 576.23)	508.58 (447.9 to 571.61)	536.01 (484.93 to 595.97)	528.61 (504.57 to 551.96)	312.84 (292.93 to 328.97)	607.69 (559.18 to 681.04)	355.36 (302.58 to 417.4)

	MCADD	MSUD	IVA	GA1	PKU	HCU	CHT	SCD - affected	SCD - carrier	Cf - affected	Cf - carrier
**Basecase**											
IP - teleconsultation	435.21 (374.67 to 491.31)	451.37 (383.46 to 513.81)	444.94 (381 to 503.98)	436.9 (374.56 to 501.73)	435.5 (372.25 to 500.48)	440.29 (376.94 to 499.21)	458.96 (407.88 to 518.92)	451.56 (427.52 to 474.91)	235.79 (215.88 to 251.92)	513.33 (464.28 to 584.71)	268.26 (213.87 to 331.19)
**Hospital visit time +25%**											
SP	475.72 (411.04 to 543.69)	483.17 (414.32 to 555.53)	479.43 (411.04 to 552.56)	505.57 (422.02 to 570.49)	509.19 (422.66 to 572.25)	501.26 (417.86 to 565.93)	539.34 (484.85 to 595.21)	601.49 (540.9 to 647.45)	237.12 (190.58 to 279.04)	667.21 (620.93 to 726.61)	265.35 (196.23 to 331.83)
IP - home visit	581.39 (523.41 to 641.87)	588.67 (527.54 to 652.9)	584.93 (524.9 to 648.33)	584.93 (524.9 to 648.33)	588.9 (526.56 to 653.63)	585.99 (525.3 to 649.01)	613.42 (562.34 to 673.37)	603.32 (576.94 to 627.99)	312.84 (292.93 to 328.97)	685.09 (636.59 to 758.44)	355.36 (302.58 to 417.4)
IP - teleconsultation	512.62 (452.08 to 568.71)	528.77 (460.86 to 591.22)	522.35 (458.41 to 581.38)	514.3 (451.97 to 579.13)	512.9 (449.66 to 577.89)	517.7 (454.35 to 576.61)	536.37 (485.29 to 596.33)	526.28 (499.89 to 550.94)	235.79 (215.88 to 251.92)	590.73 (541.69 to 662.12)	268.26 (213.87 to 331.19)
**Not face-to-face external interaction time -25%**											
SP	371.19 (320.27 to 424.12)	372.05 (320.41 to 426.32)	369.25 (317.96 to 424.09)	394.05 (326.95 to 443.17)	401.96 (331.37 to 451.15)	390.82 (323.48 to 439.93)	422.17 (380.27 to 462.54)	486.43 (435.19 to 525.98)	197.31 (157.14 to 236.41)	544.71 (502.43 to 594.98)	214.99 (158.19 to 265.79)
IP - home visit	474.05 (431.13 to 519.3)	477.15 (431.3 to 525.32)	474.34 (429.32 to 521.89)	474.34 (429.32 to 521.89)	479.91 (432.89 to 527.68)	475.13 (429.62 to 522.41)	495.71 (457.4 to 540.67)	487.8 (467.66 to 506.59)	260.6 (245.66 to 272.7)	558.12 (519.56 to 616.04)	296.48 (255.04 to 342.96)
IP - teleconsultation	396.5 (351.08 to 437.94)	406.26 (355.32 to 453.09)	401.44 (353.48 to 445.71)	395.4 (348.65 to 444.03)	396.95 (349.54 to 445.24)	400.55 (351.65 to 443.43)	411.95 (373.65 to 456.92)	404.05 (383.9 to 422.84)	176.85 (161.91 to 188.94)	457.05 (418.2 to 512.28)	203.19 (161.05 to 250.19)
**Not face-to-face external interaction time +25%**											
SP	502.85 (421.57 to 588.86)	516.88 (430.81 to 607.32)	512.2 (426.72 to 603.61)	539.69 (439.52 to 620.1)	539.02 (435.93 to 618.74)	534.3 (434.38 to 614.76)	579.11 (510.31 to 651.5)	641.85 (571.63 to 695.16)	276.92 (224.5 to 322.64)	712.3 (659.18 to 783.53)	315.71 (233.98 to 399.12)
IP - home visit	611.32 (538.56 to 687.34)	622.78 (546.37 to 703.07)	618.11 (543.07 to 697.36)	618.11 (543.07 to 697.36)	620.47 (541.97 to 701.74)	619.43 (543.58 to 698.22)	653.72 (589.87 to 728.66)	644.13 (613.81 to 673.52)	365.08 (340.19 to 385.25)	734.66 (675.81 to 825.17)	414.24 (350.09 to 491.96)
IP - teleconsultation	551.33 (475.86 to 621.72)	573.88 (489 to 651.94)	565.86 (485.92 to 639.65)	555.8 (477.88 to 636.83)	551.45 (472.05 to 633.51)	557.44 (479.79 to 632.61)	583.38 (519.53 to 658.32)	573.79 (543.47 to 603.17)	294.74 (269.85 to 314.9)	647 (586.8 to 735.78)	333.33 (266.83 to 411.99)
**Not face-to-face internal interaction time -25%**											
SP	437.02 (372.34 to 504.98)	444.47 (375.61 to 516.82)	440.73 (372.34 to 513.85)	466.87 (383.31 to 531.79)	470.49 (383.95 to 533.55)	462.56 (379.15 to 527.23)	500.64 (446.15 to 556.51)	564.14 (504.36 to 609.83)	237.12 (190.58 to 279.04)	628.51 (582.22 to 687.9)	265.35 (196.23 to 331.83)

	MCADD	MSUD	IVA	GA1	PKU	HCU	CHT	SCD - affected	SCD - carrier	Cf - affected	Cf - carrier
**Basecase**											
IP - home visit	542.68 (484.71 to 603.17)	549.97 (488.83 to 614.19)	546.23 (486.19 to 609.63)	546.23 (486.19 to 609.63)	550.19 (487.85 to 614.93)	547.28 (486.6 to 610.31)	574.71 (523.64 to 634.67)	565.97 (540.73 to 590.04)	312.84 (292.93 to 328.97)	646.39 (597.88 to 719.74)	355.36 (302.58 to 417.4)
IP - teleconsultation	473.91 (413.37 to 530.01)	490.07 (422.16 to 552.52)	483.65 (419.7 to 542.68)	475.6 (413.26 to 540.43)	474.2 (410.95 to 539.18)	479 (415.65 to 537.91)	497.67 (446.59 to 557.62)	488.92 (463.69 to 513)	235.79 (215.88 to 251.92)	552.03 (502.98 to 623.42)	268.26 (213.87 to 331.19)
**Not face-to-face internal interaction time +25%**											
SP	437.02 (372.34 to 504.98)	444.47 (375.61 to 516.82)	440.73 (372.34 to 513.85)	466.87 (383.31 to 531.79)	470.49 (383.95 to 533.55)	462.56 (379.15 to 527.23)	500.64 (446.15 to 556.51)	564.14 (504.36 to 609.83)	237.12 (190.58 to 279.04)	628.51 (582.22 to 687.9)	265.35 (196.23 to 331.83)
IP - home visit	542.68 (484.71 to 603.17)	549.97 (488.83 to 614.19)	546.23 (486.19 to 609.63)	546.23 (486.19 to 609.63)	550.19 (487.85 to 614.93)	547.28 (486.6 to 610.31)	574.71 (523.64 to 634.67)	565.97 (540.73 to 590.04)	312.84 (292.93 to 328.97)	646.39 (597.88 to 719.74)	355.36 (302.58 to 417.4)
IP - teleconsultation	473.91 (413.37 to 530.01)	490.07 (422.16 to 552.52)	483.65 (419.7 to 542.68)	475.6 (413.26 to 540.43)	474.2 (410.95 to 539.18)	479 (415.65 to 537.91)	497.67 (446.59 to 557.62)	488.92 (463.69 to 513)	235.79 (215.88 to 251.92)	552.03 (502.98 to 623.42)	268.26 (213.87 to 331.19)
